# Navigating Substance Use Care in the Emergency Department: A Scoping Review

**DOI:** 10.1016/j.acepjo.2025.100318

**Published:** 2026-02-06

**Authors:** Aneeqah H. Naeem, Jhanavi Kapadia, Jon Soske, Raina Zhang, Tobias Kirchwey, Misha Fan, Sara J. Becker, Charlie Merrick, Georgia R. Goodman, Elizabeth A. Samuels

**Affiliations:** 1 Warren Alpert Medical School of Brown University, Providence, Rhode Island, USA; 2 The University of New England College of Osteopathic Medicine, Biddeford, Maine, USA; 3 Department of Emergency Medicine, Warren Alpert Medical School of Brown University, Providence, Rhode Island, USA; 4 Department of Infectious Disease, The Miriam Hospital, Providence, Rhode Island, USA; 5 Department of Emergency Medicine, David Geffen School of Medicine, University of California, Los Angeles, California, USA; 6 Department of Emergency Medicine, SUNY Downstate, Brooklyn, New York, USA; 7 Brown University, Providence, Rhode Island, USA; 8 Department of Psychiatry and Behavioral Sciences, Feinberg School of Medicine, Northwestern University, Chicago, Illinois, USA

**Keywords:** patient navigation, peer recovery, addiction treatment, substance use disorder

## Abstract

**Objectives:**

Emergency departments (ED) are increasingly implementing community health worker (CHW) and peer recovery specialist (PRS) programs to engage patients with substance use disorders and link them to harm reduction and addiction treatment services. This scoping review aims to provide a broad overview of current research on ED-based CHW and PRS programs in North America.

**Methods:**

We conducted a comprehensive database search on PubMed, Embase, MEDLINE/OVID, and World of Science through December 31, 2022. Articles were screened for inclusion by 2 reviewers with conflicts resolved by a third reviewer. Data extraction from full-text articles was completed by 3 reviewers and checked for accuracy by a separate reviewer.

**Results:**

We screened 12,187 abstracts, reviewed 398 full-text articles, and selected 64 papers about 35 distinct programs for inclusion. Study design included a mix of retrospective and prospective analyses, including a few randomized control trials. Core program components include services navigation (97.1%), motivational interviewing and/or a brief intervention (65.7%), harm reduction services (42.9%), and case management (28.6%). Most programs evaluating addiction treatment engagement noted comparative increase in treatment engagement after program implementation, which includes initiation of medication for opioid use disorder and outpatient treatment engagement. Programs evaluating ED utilization noted a reduction in ED visits after program implementation. Comparative outcome research about overdose, hospitalizations, mortality, and cost were limited.

**Conclusion:**

ED-based CHW and PRS programs varied in design and services provided. Most studies measuring engagement reported favorable effects, although with limited effect size and heterogenous study quality, design, and metrics that limit generalizability. Future research should evaluate program effectiveness and identify key elements of successful ED-based programs to inform implementation.

## 1 Introduction

The emergency department (ED) is on the front lines of caring for patients with substance use disorders (SUDs) and is an important entry point for SUD treatment engagement and services linkage. ED interventions to reduce alcohol- and drug-related harms include brief negotiated interviewing, behavioral counseling, naloxone distribution, addiction treatment initiation, and patient navigation for linkage to community services.^1^

Community health worker (CHW) and peer recovery specialist (PRS) programs for people with SUDs have been implemented and studied in community settings since 1997. A CHW is a frontline public health worker who identifies with the communities that they serve and acts as liaison between the health care system and the community.^2^ PRSs are individuals with a lived experience of SUD trained to provide nonclinical support and services navigation.^3^ Although these roles have distinct histories and definitions, in practice they often blend together, with individuals trained for one role working in the other, moving back and forth between roles, or combining aspects of both roles in practice.^4^

Over the last decade there has been increased implementation of CHW and PRS programs in EDs to engage ED patients with SUDs in evidence-based harm reduction, addiction treatment, and recovery services.^3^ Research about program effectiveness has been variable and the heterogeneity of evidence quality, program design, and patient populations makes it difficult to generalize research evidence.^4^, ^5^, ^6^, ^7^ We conducted a scoping review of CHW and PRS programs for ED patients with SUDs to (1) describe the current CHW and PRS program components, (2) assess evidence about program impact on treatment engagement, and (3) identify research and evidence gaps.

## 2 Methods

We conducted a scoping review of ED-based CHW and PRS programs for patients with SUDs following PRISMA guidelines (Appendix A).^8^ A protocol for this review was developed a priori but was not published. The review was initially registered as a systematic review but not as a scoping review. The full-search strategies are included in Appendix B. Studies about ED-based programs that utilized a CHW or PRS to engage ED patients with an alcohol or other SUD published in the English language were included in the review. We included studies published through 2022, written in English, and based in the United States or Canada (Table 1). Programs focused on a single SUD (eg, opioids or alcohol) or multiple SUDs were both included. Although not reflected in the literature, many of these programs have evolved to serve patients with multiple SUDs, and these programs share core components (eg, CHW/peer engagement, care navigation, and linkage to treatment) regardless of substance use. As a descriptive review, programs were summarized together rather than compared for effectiveness. For the purposes of this review, a program was defined as an intervention developed by the same team of researchers, although the intervention itself may include several sites. We included studies of interventions based primarily in the ED, even if they included community or in-hospital support. Studies that were solely based in the community or on an inpatient hospital service were excluded. A full list of inclusion and exclusion criteria are listed in Table 1.

**Table 1:** Scoping review inclusion and exclusion criteria.

Category	Inclusion criteria	Exclusion criteria
Setting	Program based at least partially in the emergency department (ED)	Program solely based in the community or inpatient setting
		Papers published outside of the US or Canada
Personnel	Utilized peer support workers or community health workers (CHWs)	Intervention staff not specified as CHWs or peers
		Only non-CHW/peer staff delivered services
		CHWs/peers only involved in research data collection (not intervention delivery)
Substance use focus	Focused on substance use disorder (including alcohol, opioids, or marijuana)	Intervention focused only on tobacco use
Modality	Included an in-person component	Intervention only digital with no in-person component
Geography	Program located in the United States or Canada	
Publication	Written in English	
	Published through the end of 2022	

With assistance from a medical librarian, we conducted a database search of the literature on PubMed, Embase, MEDLINE/OVID, and World of Science. Search terms used are in Appendix B. The initial search across all databases was initially conducted on June 28, 2021. A repeat search was conducted prior to analysis to include all articles published through December 31, 2022.

All identified articles were uploaded to Covidence, a web-based review platform, and duplicate articles were removed. Articles were initially screened individually by AHN, JK, TK, MF, and RZ based on title and abstract. All conflicts were resolved by EAS. Following an initial screen, all selected articles underwent a full-text review by AHN under the supervision of EAS. The finalized list was reviewed by AHN and EAS to ensure each article met inclusion criteria. All reviews included in the initial search were reviewed for any articles not included in the search by AHN to ensure no articles were missed.

Data elements to be extracted were identified *a priori.* Data were extracted by AHN, JK, and RZ from each of the articles using a standardized data extraction form which included study characteristics (including size, location, type of study), intervention details (description of intervention, screening modality, primary person conducting screening, intervention team members), and outcomes measured (treatment engagement, hospitalizations, overdoses, mortality, ED visits, patient-reported outcomes, health-related social needs). Questions or ambiguities in data were discussed and agreed on by consensus between AHN and EAS. Strength of evidence was not assessed as part of this scoping review, given the highly variable study data and designs ranging from descriptive assessments to randomized control trials. Each program was contacted by AHN to ensure accuracy of intervention program components. Extracted data were synthesized using descriptive statistics and narrative synthesis, with programs grouped and summarized by study design, intervention characteristics, and reported outcomes.

## 3 Results

**Figure 1:**
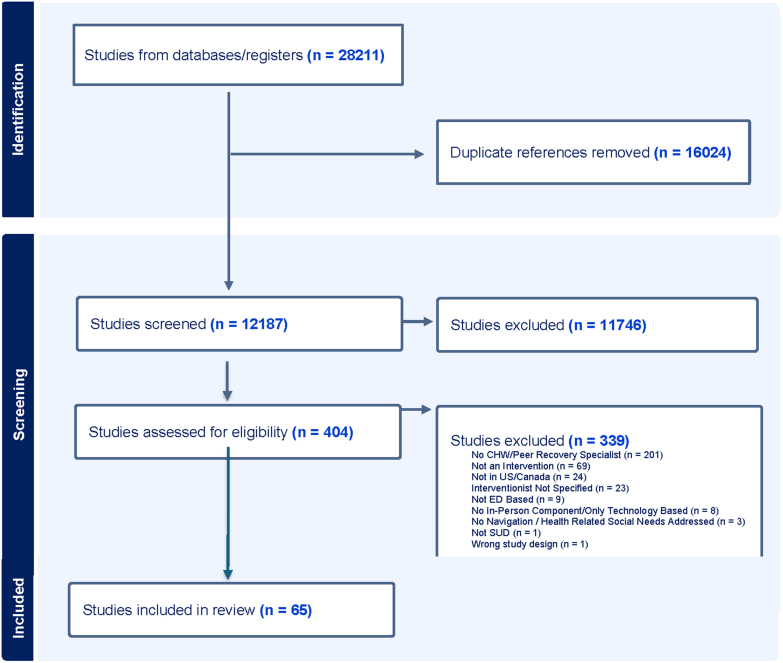
Scoping review flow chart.

Our search identified 28,665 papers and abstracts, of which 16,253 were removed as duplicates (Fig. 1). In total, 12,408 abstracts were screened, 441 were assessed for full-text review, and 64 papers were included in the review. The 64 papers described a total of 35 distinct programs. Thirty papers were retrospective studies and 34 were prospective. Nearly 3 quarters of studies included (70.3%, 45/64) were peer-reviewed manuscripts; the remaining were conference abstracts. Most studies were observational (70.3%, 45/64), 14 were randomized control trials (RCTs) (21.9%, 14/64), and 6 of the 64 (9.4%) were qualitative studies. Table 2 presents detailed information on each included study,^9^, ^10^, ^11^, ^12^, ^13^, ^14^, ^15^, ^16^, ^17^, ^18^, ^19^, ^20^, ^21^, ^22^, ^23^, ^24^, ^25^, ^26^, ^27^, ^28^, ^29^, ^30^, ^31^, ^32^, ^33^, ^34^, ^35^, ^36^, ^37^, ^38^, ^39^, ^40^, ^41^, ^42^, ^43^, ^44^, ^45^, ^46^, ^47^, ^48^, ^49^, ^50^, ^51^, ^52^, ^53^, ^54^, ^55^, ^56^, ^57^, ^58^, ^59^, ^60^, ^61^, ^62^, ^63^, ^64^, ^65^, ^66^, ^67^, ^68^, ^69^, ^70^, ^71^^,^^83^ and Table 3 presents a summary of demographic information. Key extracted elements are elaborated in the following sections.

**Table 2:** Characteristics of patient navigation programs and included research studies.

Program name	Papers associated	Study design (prospective vs retrospective + design)	Study size (participants)	Number of ED sites	Region	Geographic setting	Hospital type	Program targeted at a specific substance?	People on the intervention team	ED only vs ± inpatient
California (CA) Bridge	Snyder et al^9^	Retrospective, pilot	12,009	52	West	Urban/suburban, rural	Academic, community	Yes, opioids	Physician, substance use navigator	ED only
	Anderson et al^10^	Retrospective, cohort	1328	3	West	Urban/suburban	Academic, community	No		
ED-LINC	Whiteside et al^11^	Prospective, RCT	40	1	West	Urban	Academic	Yes, opioids	Physician, social worker/licensed mental health counselor, care manager, psychiatrist	ED only
LA Hospital	Kunz et al^12^	Prospective, RCT	294	1	West	Urban	NA/NS	Yes, Alcohol	Health promotion advocate	ED only
	Bazargan-Hejazi et al^13^	Prospective, pilot	295							
Nevada Mobile Recovery Outreach Team	Wagner et al^14^	Mixed methods, quantitative/qualitative	25 providers	6	West	Urban/suburban, rural	Academic, community	Yes, opioids	Physician, peer recovery coach, state-licensed/certified alcohol and drug counselors	ED only
	Wagner et al^15^	Prospective, qualitative	30							
	Smith et al^16^	Prospective, RCT	829 (estimated)							
--	McPadden et al^17^	Prospective, cohort	113	3	West	Urban	NA/NS	No	Health care navigators, medical case managers, medical teams	ED + in-patient/community
--	Moulin et al^18^	Prospective, cohort	294	1	West	Urban	Academic	No	Physician, certified alcohol and drug abuse counselor	ED only
--	Reed et al^19^	Prospective, RCT	791	1	West	Urban	Military	Yes, alcohol	Health educator	ED only
Indiana RCS	Staton et al^20^	Retrospective, qualitative	NA/NS	9	Midwest	NA/NS	NA/NS	Yes, opioids	Peer recovery coach	ED + in-patient/community
Project POINT	McGuire et al^21^	Prospective, report	NA/NS	1	Midwest	NA/NS	NA/NS	Yes, opioids	Peer recovery coach, social worker	ED + in-patient/community
	Watson et al^83^	Prospective, pilot	70			Urban	Academic			
	Watson et al^22^	Retrospective, Quasi-experimental	1462			Urban	NA/NS			
ROOT	Dahlem et al^23^	Retrospective, pilot	122	2	Midwest	NA	Academic	Yes, opioids	Peer recovery coach, case management navigator	ED + in-patient/community
--	Lewandowski et al^24^	Retrospective, report	148	1	Midwest	Urban	Academic	Yes, opioids	Peer recovery coach	ED only
--	Schulz et al^25^	Retrospective, qualitative	15 staff	1	Midwest	NA/NS	Community	No	Physician, nurse, peer recovery coach, behavioral health crisis clinicians, clinical community team	ED + in-patient/community
CHSURP	Monico et al^26^	Retrospective, report	79,899	23	South	NA/NS	NA/NS	No	Nurse, peer recovery coach	ED + in-patient/community
FAVOR	Carey et al^27^	Prospective, cohort	82	1	South	Urban	NA/NS	Yes, opioids	Peer recovery coach	ED + in-patient/community
	Fabiano et al^28^	Prospective, cohort	150							
Promotores	Cherpitel et al^29^	Prospective, RCT	698	1	South	Urban	Academic	Yes, alcohol	CHW (“Promotores”)	ED only
	Woolard et al^30^	Prospective, RCT								
	Nayak et al^31^	Prospective, RCT								
	Cherpitel et al^32^	Prospective, RCT								
	Ramirez et al^33^	Prospective, RCT								
	Bernstein et al^34^	Prospective, RCT								
--	Bogan et al^35^	Prospective, pilot	241	3	South	NA/NS	Academic, community	Yes, opioids	Physician, nurse, social worker/licensed mental health counselor, patient navigator	ED + in-patient/community
--	Gertner et al^36^	Prospective, report	260	1	South	Urban	Academic	Yes, opioids	Physician, peer support specialist, case manager, pharmacist	ED only
--	Jennings et al^37^	Prospective, cohort	522	5	South	NA/NS	Academic, community	No	Peer recovery specialist	ED only
--	Johnson, et al^38^	Prospective, report	NA/NS	1	South	NA/NS	NA/NS	No	Health education specialist	ED only
--	Kaur and Melville^39^	Prospective, pilot	4166	1	South	NA/NS	NA/NS	No	Peer support specialist, licensed addiction medicine professional	ED + in-patient/community
--	Ware et al^40^	Retrospective, report	666	1	South	Urban	Academic	No	Triage nurse, peer recovery coach	ED
--	Webb et al^41^	Retrospective, observational cohort	785	1	South	Urban	Academic	No	Physician, nurse, peer support specialist, licensed clinical counselor	ED + in-patient/community
Boston PED	Bernstein et al^42^	Mixed methods, qualitative + one group pre-post	4899	1–7∗ ∗ originally 1 ED, expanded to 7	Northeast	Urban/suburban, rural	Academic	No∗ ∗program began with alcohol specific, then marijuana specific, and expanded	Peer recovery coach	ED only
	Bernstein et al^43^	Prospective, RCT	210							
	Bernstein et al^44^	Prospective, RCT	853							
	Bernstein et al^45^	Retrospective, pilot	2149							
HOST Program	Beauchamp et al^46^	Retrospective, report	1363	1	Northeast	NA/NS	Academic	No	Physician, social worker/licensed mental health counselor, certified recovery specialist, social worker, hospital opioid support team (host), medical toxicologists	ED + in-patient/community
	Beauchamp et al^47^	Prospective, report	1834							
Linking MATTERS	Sokol et al^48^	Retrospective, qualitative	NA/NS	1	Northeast	NA/NS	Community	Yes, opioids	Physician, nurse, social worker/licensed mental health counselor, peer recovery coach, physician assistant	ED + in-patient/community
LOOP	Samuels^49^	Retrospective, report	NA/NS	2 - 3∗ ∗ papers included 2 or 3 ED sites	Northeast	Urban/suburban	Academic	Yes, Opioids	Peer recovery specialist, physician, nurse, social worker/licensed mental health counselor	ED + in-patient/community
	Samuels et al^50^	Retrospective, one group pre-post	856				Academic, community			
	Samuels et al^51^	Retrospective, observational cohort	151				Academic			
	Goedel et al^52^	Prospective, RCT	650				Academic			
	Samuels et al^53^	Retrospective, one group pre-post	555				Academic, community			
	Waye et al^54^	Retrospective, cross-sectional	1392				Academic, community			
	Jacka et al^55^	Retrospective, cohort	742				Academic, community			
	Lawrence et al^56^	Retrospective, cohort	783				Academic, community			
	Reddy et al^57^	Retrospective, cross-sectional	734				Academic			
	Samuels et al^58^	Retrospective, one group pre-post	1585				Academic, community			
	Beaudoin et al^59^	Prospective, RCT	648				Academic, community			
New Jersey opioid overdose recovery program	Liebling et al^60^	Retrospective, cohort	30,263 patient visits	18	Northeast	Urban/suburban	Academic, community	No	Patient navigator, recovery specialist	ED + in-patient/community
Project ASSERT	Bernstein et al^61^	Prospective, one group pre-post	1096	1	Northeast	Urban	Academic	No	Health promotion advocate/licensed drug and alcohol counselors	ED only
	D'Onofrio and Degutis^62^	Retrospective, report	22,534 (screened)							
	Faiz et al^63^	Retrospective, cohort	782							
Project NYSBIRT-II	Auerbach et al^64^	Prospective, report	26,711 (pre-screened), 2270 (screened)	4	Northeast	Urban	NA/NS	No	Nurse, health coach	ED only
	Kapoor et al^65^	Prospective, report	53,936 (prescreen), 4175 (full screen)							
Project Connect	Harrison and Morgenstern^66^	Prospective, one group pre-post	298	2	Northeast	NA/NS	NA/NS	No	Peer recovery coach	ED only
Relay	Welch et al^67^	Prospective, report	649	7	Northeast	Urban	NA/NS	Yes, opioids	Peer recovery coach	ED + in-patient/community
--	Khan et al^68^	Retrospective, pilot	1049	1	Northeast	NA/NS	NA/NS	No	Physician, certified addiction recovery coaches, certified recovery peer advocates	ED only
--	Lowenstein et al^71^	Retrospective, one group pre-post	2665	3	Northeast	Urban	Academic	Yes, opioids	Physician, peer recovery coach	ED only
--	Lukacs^69^	Retrospective, pilot	448	1	Northeast	Suburban	Community	No	Social worker/licensed mental health counselor, peer recovery coach	ED only
--	Ramdin et al^70^	Retrospective, one group pre-post	NA/NS	1	Northeast	Urban	NA/NS	Yes, opioids	Peer recovery coach	ED only

NA/NS, not applicable/not specified; ED, emergency department; RCT, randdomized controllled trial; PED, pedatric emergency department.

**Table 3:** Emergency department substance use disorder patient navigation program characteristics.

Category	Subcategory	Number of programs (%)
Location	West	7 (20.0%)
	Midwest	5 (14.3%)
	South	10 (28.6%)
	Northeast	13 (37.1%)
Program size∗	0–500 participants	12 (34.2%)
	501+ participants	19 (54.3%)
Substance use disorder focus	Alcohol	3 (8.6%)
	Opioid	15 (42.9%)
	Any/not specified	17 (48.6%)

Not all programs reported participant count.

∗ If multiple sample sizes were reported for a single program, the largest was used.

### 3.1 Program Descriptions

#### 3.1.1 Study and program size

Study size ranged from small cohort studies at single sites (30 participants)^14^ to large, multisite studies that screened and delivered brief interventions to large volumes of patients (79,899 brief interventions).^26^ Of the 35 unique programs, program size ranged from 1 to a maximum of 52 sites. Over half of programs (57.1%, 20/35) were in urban areas, none exclusively in rural areas, and 3/35 (8.6%) were in both urban and rural hospitals. About half (51.4%, 18/35) of programs were substance specific, primarily opioid (42.9%, 15/35), and 3 of 35 (8.6%) were specific to patients with alcohol use disorder.

#### 3.1.2 Program staff composition

Intervention team composition varied widely. In addition to the CHW/PRS, nearly 40% of teams included a physician or advanced practice practitioner (13/35), about a quarter (25.7%, 9/35) included a nurse, and less than a quarter included a social worker/licensed mental health counselor (20.0%, 7/35). Terminology used to refer to the peer support worker varied, including peer recovery coach or specialist (45.7%, 16/35), patient navigators (8.6%, 3/35), health promotion advocates (5.7%, 2/35), substance use navigator (2.9%%, 1/35), and community health worker (2.9%, 1/35). More than half of the programs (57.1%, 20/35) worked in the ED only, and the other portion (42.9%, 16/35) also worked in community and inpatient settings.

#### 3.1.1 Program features

**Figure 2:**
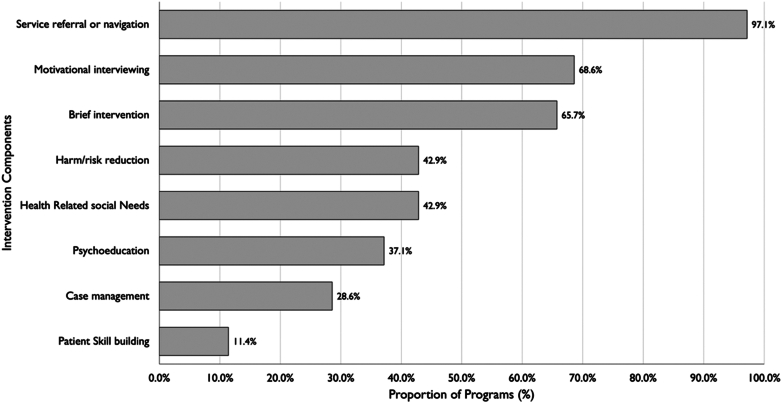
Core components of emergency department patient navigator and community health worker programs for patients with substance use disorder.

Core program components (Fig. 2) include services navigation (97.1%, 34/35), motivational interviewing (68.6%, 24/35), and/or a brief intervention (65.7%, 23/35), harm or risk reduction services (42.9%, 15/35, such as naloxone or sterile consumption supplies), psychoeducation (37.1%, 13/35), case management (28.6%, 10/35), and addressing patients’ health-related social needs (42.9%, 15/35). Studies often did not specify how they operationalized or defined some of these services, complicating comparison across studies.

### 3.2 Study Outcomes

Study outcomes measured included harm reduction and treatment services provision and engagement (26/35), equity in services delivery (4/35), changes in substance use (7/35), overdose (4/35), ED utilization (7/35), hospitalizations (5/35), death (4/35), and cost (4/35).

#### 3.2.1 Naloxone distribution

Seven studies reported data related to naloxone distribution. Overall, programs showed a marked increase in naloxone distribution after program implementation in both statewide programs and single-site initiatives.^23^^,^^35^^,^^55^^,^^58^^,^^70^

#### 3.2.2 Addiction treatment engagement

Engagement in evidence-based addiction treatment is a critical service ED CHW and PRS programs provide. Although many programs included treatment engagement as part of the intervention designs, only 26 studies, analyzing 19 programs, reported initiation of medications for opioid use disorder (MOUD; buprenorphine or methadone) or treatment referral (inpatient, outpatient, detox programs) (Table 4).^9^, ^10^, ^11^^,^^18^^,^^22^^,^^24^^,^^26^^,^^35^, ^36^, ^37^, ^38^^,^^40^^,^^41^^,^^47^^,^^50^^,^^51^^,^^53^^,^^55^^,^^57^, ^58^, ^59^^,^^66^, ^67^, ^68^^,^^70^^,^^71^ Seven of 9 programs assessing changes in treatment engagement (77.8%) found an increase in treatment engagement after program implementation. MOUD administration in the ED was studied in 11 programs, with 7 studies using a comparison group. Among these 7, 5 showed a statistically significant increase in MOUD administration ranging from a 12.5% to 22.7% increase.^10^^,^^22^^,^^47^^,^^70^^,^^71^ Six programs examined attendance at follow-up appointments, with only one utilizing a control group.^9^^,^^10^^,^^26^^,^^35^^,^^37^^,^^47^^,^^67^ Anderson et al^10^ reported a 35.7% increase in attendance with engagement with a patient navigator at follow-up within 7 days after discharge (8.0% [97/1,209] versus 43.7% [52/119]) and a 34.5% increase in attendance 30 days after discharge (15.9% [192/1,209] versus 50.4% [60/119]).

**Table 4:** Emergency department substance use disorder patient navigation programs and addiction treatment engagement.

Paper	Study design	Effect	Treatment outcome
Anderson et al^10^	Retrospective, quasi-experimental	↑	• 22.7% increase in medications administered for SUD (16.8% [203/1209] vs 39.5% [47/119]), 95% CI (13.7 to 31.7) • 26.4% increase in medications prescribed for SUD (20.7% [250/1209] vs 47.1% [56/119]), 95% CI (17.1 to 35.6) • 35.7% increase in attendance at follow-up within 7 days after discharge (8.0% [97/1209] vs 43.7% [52/119]), 95% CI (26.6 to 44.7) • 34.5% increase in attendance at follow-up within 30 days after discharge (15.9% [192/1209] vs 50.4% [60/119]), 95% CI (25.3 to 43.8) • 50% (60/119) Substance use navigator engaged in treatment in 30 days of ED discharge versus no substance use navigator (15.9%, 192/1209)
Beauchamp et al^47^	Retrospective, cohort	↑	• Proportion of MOUD initiation increased from 67.5% (83/123) to 80% (140/175) from the first to last 9 months of implementation for patients engaged by addiction recovery specialist, *p* = .01 • A higher proportion of individuals engaged by the addiction recovery specialist (ARS) 243/298 (81.5%) were linked to treatment compared with people engaged by the hospital opioid support team 676/1536 (44.0%) (*p* < .001)
Beaudoin et al^59^	RCT	↔	Receipt of SUD treatment showed no difference, 32% (103/323) of patients from certified peer recovery specialist received SUD treatment versus 30% (98/325) from the control group (hospital clinical social worker)
Lowenstein et al^71^	Retrospective, pre-post	↑	• All MOUD initiation increased from 2.6% (35/1326) to 22.6% (302/1339), (delta 20%, 95% CI [12.9 to 27.1]) • Total buprenorphine increased from 1.5% (20/1326) to 21.2% (282/1339), (delta 19.6%, CI 13.1 to 26.0) • Buprenorphine given in ED increased from 1.2% (16/1326) to 14.6% (195/1339), (delta 13.4%, 95% CI (6.1 to 20.7) • Methadone given in ED had no difference, (delta 0.4%, 95% CI (−7.1 to 7.9) • Buprenorphine prescribed at discharge increased from 0.5% (7/1339) to 15.1% (202/1339), (delta 13.6%, 95% CI [6.5 to 20.7])
Ramdin et al^70^	Retrospective, pre-post	↑	Buprenorphine administration increased by 22.4% (from 643 to 787), *p* = .007
Samuels et al^50^	Retrospective, pre-post	↑	Linkage to treatment increase from 12.3% (40/322) to 15.5% (50/322), *p* value < .001
Samuels et al^51^	Observational, retrospective cohort	↔	• No difference in MOUD initiation • Among people who received peer services, nonsignificant shorter time to initiation of MOUD of 81.5 days [95% CI 24, 152] vs 107 days [95% CI 53, 247])
Samuels et al^53^	Retrospective, pre-post	↑	Discharge with referral to treatment increased from 9.16% (12/131) to 20.74% (78/376), *p* = .003
Samuels et al^58^	Retrospective, interrupted time series analysis	↑	Statistically significant increase in referrals for MOUD from 2.5% to 17.8% (OR 8.60 [3.78, 19.57]) and at level 1 hospitals in particular (RR 7.73 [95% CI 3.22 to 18.55]
Watson et al^22^	Retrospective, quasi-experimental difference-in-difference	↑	MOUD prescriptions increased with significant improvement (difference-in-differences estimate 1.53 [SD 0.21], *p* < .001)
Whiteside et al^11^	Pilot RCT	↔	30% (6/20) started buprenorphine in the control group, 50% (10/20) in the intervention group, but not a statistically significant difference given small sample size 95% CI (−0.10, 0.50)
Observational studies			
Bogan et al^35^	Retrospective, observational cohort	---	• 45.0% (241/535) of eligible patients received buprenorphine in the ED • 78% (187/241) of patients started on buprenorphine attending next day appointment • 46% (111/241) of patients remained in treatment 30 days later
Harrison et al^66^	Pilot	---	Of 200 patients that received navigation, 53% (106) engaged in treatment at baseline, 65% (130) at 30 days, 53% (106) at 90 days, 71% (142) at 120 days.
Jacka et al^55^	Retrospective, cohort	---	• Treatment referral rates increased from 56% to 80% (no comparison group) and were maintained over study period. • Patients 2.29 more likely to receive behavioral counseling in treatment teams with residents/APPs combined with attending physicians. (95% CI 1.68, 3.12)
Jennings et al^37^	Prospective, observational cohort	---	• 77% of patients (402/522) attended follow-up appointment, of those, 43.1% (173/402) remained in treatment at 30 days • 37.5% (196/522) received buprenorphine in the ED at the private teaching hospital, 32.4% (169/522) given buprenorphine at the academic medical centers and 30.1% (157/522) at community medical centers. • Only 10/522 patients (1.9%) received buprenorphine prescription in the ED
Johnson et al^38^	Retrospective chart review	---	Number of patients receiving treatment 1 week after ED visit increased from 19.3% to 34.4% (no comparison group)
Khan et al^68^	Pilot	---	12.4% of patients (130/1049) received handoffs to SUD providers
Lewandowski et al^24^	Retrospective chart review	---	• 78 patients given buprenorphine for OUD • 51/78 (65%) referred to outpatient programs, 27/78 (35%) referred to inpatient.
Liebling et al^60^	Implementation pilot	---	Of 4496 referrals given by patient navigators, 23.9% (1120) were to self-help groups, 23.6% (1106) were for withdrawal management, 15.4% (722) were to outpatient SUD treatment, 13.9% (652) to inpatient SUD treatment, and 8.5% (397) were to OTP or office-based OUD treatment.
Monico et al^26^	Retrospective, observational cohort	---	• Among people referred to treatment, 38.3% (6113/15,961) were successfully linked to treatment • Of the 950 patients experiencing opioid withdrawal symptoms, 70.1% (666) were administered buprenorphine in the ED • Of patients given buprenorphine in ED, 94.6% (630/666) accepted referral to outpatient buprenorphine treatment and 64.6% (430/666) attended a follow-up outpatient buprenorphine visit
Moulin et al^18^	Prospective, longitudinal	---	14% (42/294) of patients had rehabilitation and recovery placement (no comparison group)
Reddy et al^57^	Observational, cross-sectional	---	No difference in treatment referral based on race, gender, or ethnicity, *p* = 0.954
Snyder et al^9^	Retrospective chart review	---	• 59.8% (7179/12,009) of patients with OUD were administered buprenorphine • 45.1% (5414/12,009) of patients with OUD were prescribed buprenorphine • 40.1% (4818/12,009) of patients with OUD attended an OUD follow-up appointment
Ware et al^40^	Retrospective, observational cohort	---	• Medicaid enrollees more likely to be successfully linked to treatment (OR 2.94), p < .001) • Patients with AUD more likely to be successfully linked to treatment (OR 1.59), *p* = .02
Webb et al^41^	Retrospective, observational cohort	---	• White patients more likely to enroll in treatment than Black patients (aOR 1.93, 95% CI [1.11 to 2.34]), p = .003 • White patients were more likely to be transported to treatment facility than Black patients (aOR 1.50, 95% CI [1.00 to 2.23]), *p* =.049
Welch et al^67^	Retrospective chart review	---	• 165/649 (25.4%) patients accepted referrals to harm reduction • 72/649 (11.1%) patients were referred to treatment. Of these, 53% to 79% kept appointments

^↑^Comparative increase in addition treatment engagement; ↔, no comparative change in addition treatment engagement; ---, descriptive, not-comparative assessment of addiction treatment engagement due to patient navigation; aOR, adjusted odds ratio; AUD, alcohol use disorder; CI, confidence interval; ED, emergency department; MOUD, medications for opioid use disorder; OR, odds ratio; OUD, opioid use disorder; SUD, substance use disorder.

#### 3.2.3 Equity in services delivery

Four studies examined differences in services delivery by race and ethnicity. In an analysis of Project ASSERT, Faiz et al^63^ found that Black and Hispanic patients faced lower odds of barriers to inpatient OUD detox (OR = 0.73, 95% CI, 0.54–0.995). In a subanalysis of the Lifespan Opioid Overdose Prevention (LOOP) program, Reddy et al^57^ found that there was no difference in naloxone provision and treatment referral based on race, but that Black patients received peer counseling less frequently than White and Latinx patients. This is key, as engagement with a PRS or CHW may result in improved treatment engagement. Webb et al^41^ found that White patients working with a PRS were more likely to engage in formal addiction treatment (aOR 1.93, 95% CI [1.11 to 2.34], *p* = 0.003) and be transported to a treatment facility (aOR 1.50, 95% CI [1.00 to 2.23], *p* = 0.049).

#### 3.2.4 Change in substance use

Few studies (20.0%, 7/35) examined changes in individual use of alcohol, marijuana, or opioids.^11^^,^^13^^,^^19^^,^^32^^,^^43^^,^^44^^,^^61^^,^^66^ Five programs demonstrated a statistically significant decrease in substance use among program participants. Reductions in substance use varied in both the nature of the demonstrated effect and the effect size. Of the 2 programs that evaluated patient-reported outcomes related to drinking, neither showed a significant change.^13^^,^^19^ However, Cherpitel et al^32^ demonstrated a reduction in maximum daily drinks at 3 months (5.08 drinks control group versus 3.98 drinks intervention group, *p* = 0.044).

#### 3.2.5 Overdose

Only 2 studies published during the review period compared differences in overdose between intervention and nonintervention groups.^22^^,^^51^ One study showed a slight reduction in repeat overdose ED visits among people receiving a PRS, as compared with those receiving no behavioral health intervention (14/60; 23.2% versus 13/65, 20.0%), but the study was underpowered and findings were not statistically significant.^51^

#### 3.2.6 ED utilization

Most programs measuring ED utilization found a reduction in ED visits after program implementation. Of the 5 that included an experimental design, 4 demonstrated a decrease in the number of repeat ED visits.^18^^,^^39^^,^^68^^,^^69^^,^^72^ Of those studies, the magnitude of that decrease varied greatly. Lukacs et al^69^ showed a small decrease in ED visits after implementation (mean 0.2 ED visits in 60 days), whereas Moulin et al^18^ demonstrated a 67% decrease in ED visits (from 6.4 to 2.1) 6 months after intervention implementation.

#### 3.2.7 Hospitalizations

Five studies reported data on rehospitalizations with variable results.^22^^,^^28^^,^^39^^,^^67^^,^^71^ Two studies showed no significant change in hospital admissions, whereas a pre-postobservational study from North Carolina demonstrated a 38% reduction in the number of hospitalizations following program implementation.^22^^,^^38^^,^^71^

#### 3.2.8 Death

Death was included as an outcome in 5 studies.^16^^,^^17^^,^^23^^,^^51^^,^^67^ In the 1 study utilizing a nonrandomized comparison group, mortality among individuals receiving a PRS was slightly reduced compared with no behavioral intervention, but this was not significant (2/65; 3.1% [95% CI, 0.8%, 11.7%] versus 4/60; 6.7% [95% CI, 2.5, 16.7]).^51^ Other studies did not include a control group and reported observational data. In these studies, death rates ranged from 0.46% to 4.6%,^17^^,^^23^^,^^67^ lower than the estimate rate of death for ED patients treated after an opioid overdose.^73^

#### 3.2.9 Cost

Six studies included information about program cost, reimbursement, and cost effectiveness^9^^,^^10^^,^^12^^,^^22^^,^^26^^,^^43^ which varied across programs, depending on program structure and staffing.^12^^,^^26^ Cost varied from $125,000 per year (supporting an 1.0 FTE navigator and some effort of a physician or APP clinical champion^8^) to $300,000 (initial implementation of a program with 4 peer recovery coaches^22^). One RCT of alcohol screening and brief intervention demonstrated cost benefit,^12^ but additional cost analyses are needed, which take into account reductions in ED utilization to support sustainable program implementation.

## 4 Limitations

Our study had several limitations. First, potentially relevant studies could have been missed despite efforts to ensure multiple checks at each step of the review process. Many programs may not be published in the peer-reviewed literature and therefore were not captured in this study. Given the nebulous nature of precisely defining CHWs or PRSs, it is possible some studies had similar team members that were not adequately defined or documented and thus led to their inadvertent exclusion. Program definitions may have also varied, as there were several programs we chose to group together that evolved over time (such as those under Project ASSERT), and several studies we chose to delineate that evolved from each other (such as Project POINT from the Indiana RCS study). Some programs were limited to single sites, and others were statewide initiatives. Additionally, assessment of the types of services provided was limited by what was described in the included publications. There may be services that programs provide—such as ED MOUD initiation or harm reduction services—which were not reported in the reviewed publications or were added following publication of the manuscript or abstract. This likely resulted in an underreporting of provision of these services. Variability in reporting of outcomes also limits the ability to compare programs and given the varying composition and focus (ie, some focused on alcohol use, others on opioid use), all comparisons may not be generalizable. Furthermore, given the limited and variable nature of the data, we were unable to perform an analysis of data quality and/or pursue a more comprehensive systematic review. Finally, included several conference abstracts in our review when peer-reviewed manuscripts on the program were not available. Evidence shows that abstracts frequently differ from final research reports, and therefore, data from abstracts may not accurately represent accurate program outcomes.

## 5 Discussion

ED CHW and PRS programs for patients with SUDs vary considerably in program design, scope, and outcomes reported. Programs differed in services offered, CHW/peer training, and program structure. Although not discussed in many studies, they also received different levels of funding and institutional support. Most programs focus on patient engagement and linkage to outpatient care, although a lack of operationalized definitions raises questions over whether different programs offered the same services under these and other commonly used terms, such as system navigation. Overall, a majority of studies that studied treatment engagement found improved linkage to addiction treatment. However, outcomes were mixed, study design and quality varied, and the ability to generalize is limited due to design considerations and sample size.

Heterogeneity in program design may be related to local needs and resources, and the definition, training, and certification of a PRS vary from state to state. Alavi et al^74^ conducted qualitative interviews showing that the work CHWs/PRSs do in practice can differ substantially from official program descriptions and role delineations present in state and federal guidelines. Several papers did not meet the inclusion criteria for this review as the investigators did not clearly define the role of staff as being community- or peer-based, even if their role appeared to be that of a PRS or CHW. Very few papers defined the role of a PRS or CHW, and there are several accepted definitions and primary responsibilities for these staff members. Programs also had specific names for peers or CHWs, such as “health education specialist” or “health promotion advocate,” and it was unclear if these roles differed from the general definitions of CHWs or PRSs. Similarly, motivational interviewing and services navigation were mentioned in almost every study. However, it was unclear how these activities were defined and practiced.

In general, peers incorporate knowledge drawn from a shared experience of having a SUD, along with navigating behavioral health, SUD treatment, and medical services.^75^ Drawing from this shared experience, peers may be able to more effectively engage patients and may offer care in a way that is perceived by patients as less stigmatizing. Centering patient needs is critical in this work, considering the unique stigma and barriers experienced by patients with SUD when accessing healthcare. Further work is needed to understand how peers can effectively operationalize their lived experience to successfully engage and support patients, improve patient outcomes, and create generalizable models for effective ED peer program implementation and dissemination.^76^ Little research has been conducted to identify best practices or fully understand the impact of these programs on the people working in them. Recent studies of CHW/peer programs in non-ED contexts have reported emotional exhaustion, secondary trauma, burnout, and high rates of turnover among program staff. None of the studies included in this review examined these outcomes. There is also a need to identify the critical functions and characteristics of effective ED CHW or PRS programs to aid in the implementation and dissemination of evidence-based ED CHW/PRS programs and quality improvement of existing programs. One of the challenges in comparing the effectiveness of PRSs and CHWs across studies was a clear outline of CHW/PRS roles and service delivery.

Few programs implemented rigorous and consistent training standards across multiple sites. A program that was among the most effective was also the largest, California (CA) Bridge.^3^^,^^7^^,^^9^^,^^10^ CA Bridge is a highly resourced, statewide initiative with a standardized training and program structure. CA Bridge has worked with over 80% of CA’s EDs to implement patient navigator and ED buprenorphine programs. They provided hospitals with longitudinal funding, training, and technical support throughout program implementation. This may explain the significant increase in treatment engagement with a large effect size at CA Bridge sites compared with other single-site studies.

Less than half of the programs assessed delivered evidence-based SUD services, such as ED MOUD initiation or take-home naloxone. Although more than half of the programs provided MOUD referral, only a third initiated buprenorphine or methadone in the ED. As ED buprenorphine for OUD has become more widely adopted, and some studies included are slightly older, it is possible these programs have added this service to their program. Limited provision of MOUD in ED CHW/PRS programs demonstrates a significant gap and highlights a key area for improvement. Despite the definitive evidence demonstrating the effectiveness of MOUD in treating opioid withdrawal and reducing morbidity and mortality among individuals with OUD, the implementation of MOUD uptake in EDs has been slow.^77^^,^^78^ The programs that provide ED MOUD show significant improvement in treatment receipt.^10^^,^^22^^,^^47^^,^^70^^,^^71^^,^^77^^,^^78^ CHW/PRS programs represent an important strategy to engage ED patients with OUD, fostering a culture change in the treatment of SUDs in the ED, with the goal of increasing linkage to outpatient treatment. There is a need for research to identify CHW/PRS-level barriers to facilitation of ED MOUD initiation and navigation to outpatient services, which may further impact ED MOUD uptake.

Harm reduction services are also critical, evidence-based interventions aimed at reducing drug-related harms, but few programs discussed whether or how this was incorporated into their service delivery. Strategies to promote harm reduction include distribution of naloxone, fentanyl test strips, and sterile consumption equipment in addition to linkage to community-based harm reduction programs.^79^ Provision of these services in the ED is an important strategy to reduce drug-related harms and promote patient engagement. However, less than half of the programs provided harm reduction services. Direct distribution of naloxone to patients is recognized as the best practice for ED SUD programs, as most prescriptions for naloxone given to patients in the ED are not filled.^80^^,^^81^ Limited provision of harm reduction services may be due to a variety of factors, including program capacity, funding, staff attitudes, and varying policy environments, which may prohibit or limit provision of harm reduction services.

Only half of the programs specifically addressed patients’ health-related social needs, including housing, transportation, and food insecurity.^82^ ED patients with SUD have substantial unmet social needs, which pose significant barriers to engagement in harm reduction services, addiction treatment programs, and recovery. Only 13 of the 35 programs incorporated case management, which requires more staff time and labor. The benefits of case management include more intensive, personalized navigation, and service linkage. However, this can limit the number of patients served by a program.

Some combination of referral to outpatient treatment programs in the ED and short-term case management in the community may represent a strategy to meet varying levels of patient need and support services engagement. Building relationships with outside organizations and including a community navigation component are 2 ways that programs can establish referral pathways, address patients’ health-related social needs, and support program continuation. Some ED programs had on-call PRSs or CHWs from a community program (LOOP, ROOT, NYC Relay, Nevada Mobile Recovery Outreach Team, HOST), whereas others have these workers stationed in the ED (Project ASSERT). Both represent new methods to incorporate more intensive navigation for patients with a high level of need. Future studies should explore the strength, number, and kinds of relationships between ED programs and community or other partners that increase the chance for both successful referral and ongoing engagement.

Understanding the effectiveness of these programs is critical to understanding how to best link patients to SUD treatment and harm reduction services. Most program evaluations focused on describing treatment engagement and referral, rather than outcomes, such as ED visits, hospitalizations, overdoses, and deaths. This is likely due to how new these programs are, as 35 of the papers included in the review were published after 2020. More time is required to evaluate the impact of these programs on individual outcomes as well as program-level outcomes, such as how their impact on ED culture and stigma may moderate or mediate patient outcomes. Standardizing outcomes will be an important evaluation strategy moving forward to better identify the impacts of these programs and allow a more rigorous evaluation to identify best practices for future program development and iteration.

For all outcomes, the study design was a significant limitation in evaluating program impact. Most studies were observational, with many papers describing pilot programs, limiting the internal and external validity of the current research. Although these program descriptions and observational assessments are important and instructive, there is a need for more rigorous experimental and quasi-experimental research to identify key characteristics associated with program effectiveness to inform future program development, implementation, dissemination, and quality improvement. With limited comparative data, it is challenging to fully assess the short- and long-term impact of an intervention across all outcomes.

Finally, how we measure or evaluate CHW/PRS programs is important to consider. Traditional measures of more distal outcomes, such as long-term recovery or death, may miss more proximal outcomes, such as transportation to an appointment or preventing patients from leaving the ED against medical advice. Given the range of structural obstacles and stigma faced by people with SUDs and those in recovery, CHWs may well be effective in proximal outcomes and still show no effect on a patient’s distal outcomes, which involve multiple contacts with the healthcare systems in which the CHW may not be present. Further, it seems likely that the relationship between proximal and distal outcomes is mediated or moderated by a third group of variables, which are also acted on by the CHW/PRS, such as individual self-efficacy, recovery capital, or level of stigma within an ED. If distal outcomes are conditioned on proximal and intermediate outcomes, both of which are dependent variables affected by CHW/PRS interventions, we may need more sophisticated models to study these programs.

In summary, embedding CHW and PRS programs in the ED to engage patients with SUD and link them to treatment is a promising strategy to improve engagement in evidence-based services to reduce drug-related harms. There was significant heterogeneity in program design, but most programs that measured engagement in addiction treatment services found a significant increase in engagement over time. There is a need for improvement of ED MOUD, delivery of medications for alcohol use disorder, and harm reduction services provision among these programs, but when offered, ED CHW/PRS programs improve treatment initiation and linkage. This review demonstrates the need for further incorporation of evidence-based SUD services into existing programs and rigorous study design to further examine program effectiveness.

## Author Contributions

AN and EAS conceived the study. AN developed data extraction instrument under supervision of EAS. AN, JK, RZ, TK, and MF conducted article reviews and data extraction. AN conducted data analysis. AN and EAS drafted the manuscript, and all authors contributed substantially to its revision. EAS supervised the study and takes responsibility for the paper as a whole.

## Funding and Support

This study was supported by the Centers for Disease Control and Prevention R01CE003632, the National Insitute of General Medical Sciences (P20GM125507), and an Emergency Medicine Foundation/Society for Academic Emergency Medicine Foundation Medical Student Research Grant.

## Conflict of Interest

All authors have affirmed they have no conflicts of interest to declare.
