# Supplementary material for: Navigating Substance Use Care in the Emergency Department: A Scoping Review
Source: J Am Coll Emerg Physicians Open. 2026 Feb 6;7(2):100318. doi: 10.1016/j.acepjo.2025.100318 (PMC12907702; doi:10.1016/j.acepjo.2025.100318)
Supplement: Supplementary Appendix [file mmc1.docx]

**Appendix A:** PRISMA Guidelines

|  | | | |
| --- | --- | --- | --- |
| **Section** | **Item** | **PRISMA-ScR Checklist Item** | **Reported on Page #** |
| **TITLE** |  |  |  |
| Title | 1 | Identify the report as a scoping review. | 1 |
| **ABSTRACT** |  |  |  |
| Structured summary | 2 | Provide a structured summary that includes (as applicable): background, objectives, eligibility criteria, sources of evidence, charting methods, results, and conclusions. | 2 |
| **INTRODUCTION** |  |  |  |
| Rationale | 3 | Describe the rationale for the review in the context of what is already known. Explain why the review questions/objectives lend themselves to a scoping review approach. | 3 |
| Objectives | 4 | Provide an explicit statement of the questions and objectives being addressed with reference to key elements (e.g., population, concepts, context). | 3 |
| **METHODS** |  |  |  |
| Protocol and registration | 5 | Indicate whether a review protocol exists and where it can be accessed; provide registration information if available. | 3 |
| Eligibility criteria | 6 | Specify eligibility criteria characteristics (e.g., years, language, publication status) and provide rationale. | 3, Table 1 |
| Information sources | 7 | Describe all information sources in the search and date of last search. | 3-4 |
| Search | 8 | Present the full electronic search strategy for at least one database. | Appendix B |
| Selection of sources of evidence | 9 | State the process for selecting sources of evidence (screening and eligibility). | 4 |
| Data charting process | 10 | Describe methods of charting data, including forms used, independence/duplication, and how data were confirmed. | 4 |
| Data items | 11 | List and define all variables for which data were sought; include assumptions or simplifications. | 4 |
| Critical appraisal of individual sources | 12 | If done, explain rationale and methods for critical appraisal of sources; how this was used in synthesis. | N/A (see p. 4) |
| Synthesis of results | 13 | Describe methods for summarizing charted data. | 4 |
| **RESULTS** |  |  |  |
| Selection of sources of evidence | 14 | Give numbers screened, eligible, and included; provide reasons for exclusions (ideally a flow diagram). | 4, Figure 1 |
| Characteristics of sources of evidence | 15 | Present characteristics of each source of evidence and provide citations. | 4-5, Tables 2-3 |
| Critical appraisal within sources | 16 | If done, present data from critical appraisal (see item 12). | N/A |
| Results of individual sources | 17 | Present charted data relevant to review questions for each source. | 5-7, Table 4 |
| Synthesis of results | 18 | Summarize or present charting results in relation to review questions/objectives. | 5-7 |
| **DISCUSSION** |  |  |  |
| Summary of evidence | 19 | Summarize main results (concepts, themes, types of evidence), link to review questions, and consider relevance to key groups. | 7-10 |
| Limitations | 20 | Discuss limitations of the scoping review process. | 7 |
| Conclusions | 21 | Provide general interpretation of results, implications, and/or next steps. | 10 |
| **FUNDING** |  |  |  |
| Funding | 22 | Describe sources of funding for the evidence and review itself; explain funders' roles. | 1 |

**Appendix B:** Search Strategy

| **Table B.1:** PubMed / Ovid Medline Search Strategy | | | | | |
| --- | --- | --- | --- | --- | --- |
| **Substance Use [MeSH Terms]** | **Substance Use [Text Word]** | **Screen/Interventions [MeSH Terms]** | **Interventions [Text Word]** | **Social Variables MeSH** | **Social Variables [Text Word]** |
| "Substance-Related Disorders"[MeSH Terms] OR | "substance use"[Text Word] OR | "Psychosocial Intervention"[MeSH Terms] OR | "intervention" | Social Work, Psychiatric | "health related social needs"[Text Word] OR |
| "Addiction Medicine"[MeSH Terms] OR | "substance misuse"[Text Word] OR | "Psychotherapy"[MeSH Terms] OR | "treatment initiation"[Text Word] OR | "Social Medicine"[MeSH Terms] OR | "Unmet social needs"[Text Word] OR |
| "Behavior, Addictive"[MeSH Terms] OR | "substance abuse"[Text Word] OR | "Psychoanalytic therapy"[MeSH Terms] OR | "therapy"[Text Word] OR | "Social Support"[MeSH Terms] OR | "Social determinants of health"[Text Word] OR |
| "Drug Users"[MeSH Terms] OR | "drug use"[Text Word] OR | "Crisis Intervention"[MeSH Terms] OR | "counseling"[Text Word] OR | "Social work"[MeSH Terms] OR | "housing"[Text Word] OR |
| "Opioid-Related Disorders"[MeSH Terms] OR | "addiction"[Text Word] OR | "Counseling"[MeSH Terms] OR | "navigation"[Text Word] OR | "Social Environment"[MeSH Terms] OR | "transportation"[Text Word] OR |
| "Heroin Dependence"[MeSH Terms] OR | "drug abuse"[Text Word] OR | "Motivational Interviewing"[MeSH Terms] OR | "peer support"[Text Word] OR | "Food Insecurity"[MeSH Terms] OR | "food security"[Text Word] OR |
| "Narcotic-Related Disorders"[MeSH Terms] OR | "overdose"[Text Word] OR | "Opiate Substitution Treatment"[MeSH Terms] OR | "Recovery Coach"[Text Word] OR | "Food Security"[MeSH Terms] OR | "medical legal services"[Text Word] OR |
| "Alcoholism"[MeSH Terms] OR | "opioid"[Text Word] OR | "Referral and Consultation"[MeSH Terms] OR | "Peer"[Text Word] OR | "Homeless Persons"[MeSH Terms] OR | "homeless"[Text Word] |
| "Cocaine-Related Disorders"[MeSH Terms] OR | "alcoholism"[Text Word] OR | "Psychotherapy, Brief"[MeSH Terms] OR | "addiction treatment"[Text Word] OR | "Homeless Youth"[MeSH Terms] OR |  |
| "Amphetamine-Related Disorders"[MeSH Terms] OR | "alcohol"[Text Word] OR | "Psychology"[MeSH Terms] OR | "community health worker"[Text Word] OR | "Sociological Factors"[MeSH Terms] OR |  |
| "Substance Abuse, Intravenous"[MeSH Terms] OR | "Substance Use Disorder"[Text Word] OR | "Treatment Adherence and Compliance"[MeSH Terms] OR | "brief negotiated interview"[Text Word] OR | "Socioeconomic Factors"[MeSH Terms] OR |  |
| "Substance Abuse, Oral"[MeSH Terms] OR | "cocaine"[Text Word] OR | "Interview, Psychological"[MeSH Terms] OR | "SBIRT"[Text Word] OR | "Social Determinants of Health"[MeSH Terms] OR |  |
| "Substance Abuse Detection"[MeSH Terms] OR | "stimulants"[Text Word] OR | "Mental Health Recovery"[MeSH Terms] OR | "advocate"[Text Word] OR | "Poverty"[MeSH Terms] OR |  |
| "Marijuana Abuse"[MeSH Terms] OR | "Overdose"[Text Word] | "Community Health Services"[MeSH Terms] OR | "Screening, Brief Intervention and Referral to Treatment"[Text Word] | "Legal Services"[MeSH Terms] OR |  |
| "Inhalant Abuse"[MeSH Terms] OR |  | "Community Mental Health Services"[MeSH Terms] OR |  | "Fees, Medical"[MeSH Terms] OR |  |
| "Illicit Drugs"[MeSH Terms] OR |  | "Community Health Workers"[MeSH Terms] OR |  | "Racism"[MeSH Terms] OR |  |
| "Drug Misuse"[MeSH Terms] OR |  | "Psychosocial Support Systems"[MeSH Terms] OR |  | "Unemployment"[MeSH Terms] OR |  |
| "Prescription Drug Misuse"[MeSH Terms] OR |  | "Behavior Therapy"[MeSH Terms] OR |  | "Medically Uninsured"[MeSH Terms] OR |  |
| "Dangerous Behavior"[MeSH Terms] OR |  | "Family Therapy"[MeSH Terms] OR |  | "Housing"[MeSH Terms] OR |  |
| "Naltrexone"[MeSH Terms] |  | "Cognitive Behavioral Therapy"[MeSH Terms] OR |  | "Public Housing"[MeSH Terms] OR |  |
|  |  | "Harm reduction"[MeSH Terms] OR |  | "Residence Characteristics"[MeSH Terms] OR |  |
|  |  | "Motivation"[MeSH Terms] OR |  | "Transportation of Patients"[MeSH Terms] OR |  |
|  |  | "Mentoring"[MeSH Terms] OR |  | "Employment, Supported"[MeSH Terms] OR |  |
|  |  | "Person-Centered Psychotherapy"[MeSH Terms] OR |  | "Psychosocial Functioning"[MeSH Terms] OR |  |
|  |  | "Psychotherapy, Group"[MeSH Terms] OR |  | "Health Services Accessibility"[MeSH Terms] OR |  |
|  |  | "Mindfulness"[MeSH Terms] OR |  | "Rehabilitation, Vocational"[MeSH Terms] OR |  |
|  |  | "Acceptance and Commitment Therapy"[MeSH Terms] |  | "Domestic Violence"[MeSH Terms] OR |  |
|  |  |  |  | "Emotional Abuse"[MeSH Terms] OR |  |
|  |  |  |  | "Spouse Abuse"[MeSH Terms] OR |  |
|  |  |  |  | "Minority health"[MeSH Terms] |  |

| **Table B.2:** Embase Search Strategy | | | | | | | |
| --- | --- | --- | --- | --- | --- | --- | --- |
| **EMTREE (ED)** | **TIAB (ED)** | **EMTREE (Substance Use)** | **TIAB (Substance Use)** | **EMTREE (Psy-soc-Interventions)** | **TIAB (Psy-soc-Interventions)** | **EMTREE (Soc Var)** | **TIAB (Soc Var)** |
| emergency ward' OR | ED' OR | drug dependence' OR | substance use' OR | psychosocial' OR | intervention' | social work' | health related social needs' OR |
| emergency health service' OR | ER' OR | addiction' OR | substance misuse' OR | psychosocial intervention' OR | treatment initiation' OR | social medicine' OR | Unmet social needs' OR |
| emergency care' OR | A&E' OR | addiction medicine' OR | substance abuse' OR | psychotherapy' OR | therapy' OR | social support' OR | Social determinants of health' OR |
| emergency medicine' | emergency department' OR | injection drug user' OR | drug use' OR | psychoanalytic therapy' OR | counseling' OR |  | housing' OR |
| psychiatric emergency' OR | emergency room' OR | drug use' OR | addiction' OR | crisis intervention' OR | navigation' OR | social environment' OR | transportation' OR |
| psychiatric emergency service' OR | casualty department' OR | heroin dependence' OR | drug abuse' OR | Counseling' OR | peer support' OR | food insecurity' OR | food security' OR |
| evidence-Based Emergency Medicine' OR | emergency medicine' OR | narcotic dependence' OR | overdose' OR | motivational interviewing' OR | Recovery Coach' OR | homelessness' OR | medical legal services' OR |
| pediatric emergency department' OR | emergency ward' OR | alcoholism' OR | opioid' OR | opiate substitution treatment' OR | Peer' OR | homeless persons' OR | homeless' |
| pediatric emergency department' | casualty ward' | substance abuse' OR | alcoholism' OR | patient referral' OR | addiction treatment' OR | homeless youth' OR |  |
|  |  | drug abuse screening test' OR | alcohol' OR | short term psychotherapy' OR | community health worker' OR | social aspects and related phenomena' OR |  |
|  |  | cannabis use' OR | Substance Use Disorder' OR | psychology' OR | brief negotiated interview' OR | socioeconomics' OR |  |
|  |  | prescription drug misuse' OR | cocaine' OR | patient compliance' OR | SBIRT' OR | social determinants of health' OR |  |
|  |  | cocaine dependence' OR | stimulants' OR | psychological interview' OR | advocate' OR | poverty' OR |  |
|  |  | morphine addiction' OR | Overdose' | mental health recovery' OR | Screening, Brief Intervention and Referral to Treatment' | legal service' OR |  |
|  |  | amphetamine addiction' OR |  | community care' OR |  | medical fee' OR |  |
|  |  | cannabis addiction' OR |  | community mental health' OR |  | racism' OR |  |
|  |  | behavioral addiction' OR |  | health auxiliary' OR |  | unemployment' OR |  |
|  |  | benzodiazepine dependence' OR |  | psychosocial care' OR |  | medically Uninsured' OR |  |
|  |  | drugs used in the treatment of addiction' OR |  | behavior therapy' OR |  | housing' OR |  |
|  |  | opiate addiction' OR |  | family therapy' OR |  |  |  |
|  |  | opioid use disorder' OR |  | cognitive behavioral therapy' OR |  | demography' OR |  |
|  |  | drug misuse' OR |  | harm reduction' OR |  | patient transport' OR |  |
|  |  | prescription drug misuse' OR |  | motivation' OR |  | supported employment' OR |  |
|  |  | drug abuse' OR |  | mentoring' OR |  | social psychology' OR |  |
|  |  | substance abuse' OR |  | client centered therapy' OR |  | health care access' OR |  |
|  |  | drug dependence treatment' OR |  | mindfulness' OR |  | vocational rehabilitation' OR |  |
|  |  | intravenous drug abuse' OR |  | acceptance and commitment therapy' |  | domestic violence' OR |  |
|  |  | multiple drug abuse' OR |  | psychiatric treatment' OR |  | emotional abuse' OR |  |
|  |  | drug abuse pattern' OR |  | psychological and psychiatric procedures' OR |  | partner violence' OR |  |
|  |  | inhalant abuse' OR |  | psychoanalysis' OR |  | minority health' |  |
|  |  | illicit drug' OR |  | psychoanalytic therapy in infancy and childhood' |  |  |  |
|  |  | illicit drug inhalation' OR |  | therapy' OR |  |  |  |
|  |  | naltrexone' |  | counseling' OR |  |  |  |
|  |  |  |  | psychotherapy in adolescence' OR |  |  |  |
|  |  |  |  | group therapy' OR |  |  |  |
|  |  |  |  | psychodynamic psychotherapy' OR |  |  |  |
|  |  |  |  | psychological intervention' OR |  |  |  |
|  |  |  |  | psychosocial rehabilitation' OR |  |  |  |
|  |  |  |  | psychosocial care' OR |  |  |  |
|  |  |  |  | psychological rating scale' |  |  |  |
